# PoWRKY71 is involved in *Paeonia ostii* resistance to drought stress by directly regulating light-harvesting chlorophyll a/b-binding 151 gene

**DOI:** 10.1093/hr/uhad194

**Published:** 2023-09-25

**Authors:** Yuting Luan, Zijie Chen, Ziwen Fang, Xingqi Huang, Daqiu Zhao, Jun Tao

**Affiliations:** College of Horticulture and Landscape Architecture, Yangzhou University, Yangzhou 225009, China; College of Horticulture and Landscape Architecture, Yangzhou University, Yangzhou 225009, China; College of Horticulture and Landscape Architecture, Yangzhou University, Yangzhou 225009, China; Department of Biochemistry, Purdue University, West Lafayette, IN 47907, USA; College of Horticulture and Landscape Architecture, Yangzhou University, Yangzhou 225009, China; College of Horticulture and Landscape Architecture, Yangzhou University, Yangzhou 225009, China; Joint International Research Laboratory of Agriculture and Agri-Product Safety, the Ministry of Education of China, Yangzhou University, Yangzhou 225009, China

## Abstract

Although the functions of WRKY transcription factors in drought resistance are well known, their regulatory mechanisms in response to drought by stabilising photosynthesis remain unclear. Here, a differentially expressed *PoWRKY71* gene that was highly expressed in drought-treated *Paeonia ostii* leaves was identified through transcriptome analysis. *PoWRKY71* positively responded to drought stress with significantly enhanced expression patterns and overexpressing *PoWRKY71* in tobacco greatly improved plant tolerance to drought stress, whereas silencing *PoWRKY71* in *P. ostii* resulted in a drought-intolerant phenotype. Furthermore, lower chlorophyll contents, photosynthesis, and inhibited expression of photosynthesis-related light-harvesting chlorophyll a/b-binding 151 (*CAB151*) gene were found in *PoWRKY71*-silenced *P. ostii*. Meanwhile, a homologous system indicated that drought treatment increased *PoCAB151* promoter activity. Interactive assays revealed that PoWRKY71 directly bound on the W-box element of *PoCAB151* promoter and activated its transcription. In addition, *PoCAB151* overexpressing plants demonstrated increased drought tolerance, together with significantly higher chlorophyll contents and photosynthesis, whereas these indices were dramatically lower in *PoCAB151*-silenced *P. ostii.* The above results indicated that PoWRKY71 activated the expression of *PoCAB151*, thus stabilising photosynthesis via regulating chloroplast homeostasis and chlorophyll content in *P. ostii* under drought stress. This study reveals a novel drought-resistance mechanism in plants and provides a feasible strategy for improving plant drought resistance via stabilising photosynthesis.

## Introduction

Drought is a harmful environmental constraint throughout the whole process of plant growth. In the past decade, the agricultural economic loss caused by drought stress accounted for about 30% (about US$30 billion) of the total loss [1]. For example, drought stress caused yield reduction and quality decline of rice [2], and the growing areas and production of soybean were also severely limited by water deficiency [3]. Therefore, scientists are devoted to exploring the mechanism of plant drought resistance and finding strategies for improving plant tolerance under drought stress.

Drought stress has irreversible negative effects on both the vegetative and reproductive growth of plants. Rice actively re-established a new water-absorbing system by promoting root growth under drought [4]. Moreover, plants have evolved early flowering during drought adaptation [5, 6]. All these shreds of evidence indicate that drought stress slows down the overall ecological evolution of plants. When plants persistently suffer from drought stress, they present multiple injuries including decreased net photosynthetic rate (*Pn*), increased reactive oxygen species (ROS) accumulation, cell membrane system damage, electrolytes exudation, and a collapse in the antioxidant system [3, 7]. To adapt to this external environmental stress, the plant itself has evolved the best strategy to maintain water balance and life. The physiological responses are achieved by regulating stomatal closure, root-shoot ratio, and stimulating antioxidant systems consisting of superoxide dismutase (SOD), catalase (CAT), and peroxidase (POD) [8, 9]. In a deeper molecular response, plants continue to evolve drought-responsive genes associated with drought signaling transduction and metabolite production to protect cells from damage [10, 11]. Currently, a considerable number of studies reported the essential function of abscisic acid (ABA) accumulation in plant drought resistance mechanisms, and many genes involved in ABA pathway have been identified including *Malus domestica* ABA receptor *MdPYL9* (pyrabactin resistance-like) gene [12]. Simultaneously, drought stress stimulates specific accumulation of metabolites such as proline, flavonoids, and carotenoids to prevent the destruction of plant cells and membrane systems [1, 13], while the specific molecular mechanisms are ambiguous and need to be further studied.

Drought stress triggers the expression of massive endogenous genes including effector genes encoding defense proteins or regulatory genes encoding transcription factors [3]. Numerous transcription factor families have been identified to be involved in drought dividing into NACs (NAM, ATAF1/2, CUC1/2), MYBs (myeloblastosis), bZIPs, and WRKYs [14]. Among them, WRKY transcription factors are named based on the highly conserved WRKYGQK residues and always play central roles in abiotic stress responses [7, 15]. In *Brassica napus*, the expression of most WRKY transcription factors increased under drought [16]. In *Arabidopsis thaliana*, *AtWRKY57* endowed plants with high drought tolerance [17]. Notably, WRKY transcription factors always regulate drought defense genes existing W-box ([C/T]TGAC[T/C]) elements in their promoters [18]. *Zea mays* ZmWRKY79 positively regulated drought resistance by activating *ZmAAO3* (abscisic aldehyde oxidase) gene promoter to reduce hydrogen peroxide (H_2_O_2_) and malondialdehyde (MDA) content [19]. *Manihot esculenta* MeWRKY20 interacted with MeHSP90 (heat shock protein) to form a complex that promoted *MeNCED5* (9-*cis*-epoxycarotenoid dioxygenase) transcription to help plants withstand drought stress [20]. *Populus trichocarpa PtrWRKY75* enhanced drought resistance by specifically targeting *PtrPAL1* (phenylalanine ammonia lyase) gene promoter to promote salicylic acid biosynthesis [21]. Overall, the above evidence reveals that WRKY mediates multiple pathways to mitigate drought in plants, whereas few studies have examined their regulation of drought resistance at the level of regulating light capture and transfer.

*Paeonia ostii* is a widely cultivated woody oil crop that benefits human health with 40% α-linolenic acid in its seed oil [22], while its planting expansion is often restricted by different stresses, such as high temperature [23], drought [24], waterlogging [25], and copper [26, 27]. In regard to drought stress, previous study delineated *P. ostii* physiological response to drought, and some further exogenous treatments such as ferulic acid, graphene oxide, fulvic acid, and CaCl_2_ were also applied to alleviate this stress [28–31]. Moreover, a drought-induced *CCoAOMT* (caffeoyl-CoA *O*-methyltransferase) gene was proven to optimize the lignin content of transgenic plants to withstand drought [32]. Apart from this, more molecular analysis and genetic methods related to *P. ostii* drought stress are still blank and need to be further overcome. In this study, a drought-responsive WRKY transcription factor *PoWRKY71* that showed substantially upregulated expressions in drought-treated *P. ostii* leaves was identified and cloned [24]. *PoWRKY71* played a positive role in resisting drought stress, and it directly activated *PoCAB151* transcription, which participated in stabilising photosynthesis via regulating chloroplast homeostasis and chlorophyll content. Overall, these results revealed a new pathway in plants that responds to drought stress.

## Results

### *PoWRKY71* is a positive regulator induced by drought stress

**Figure 1:**
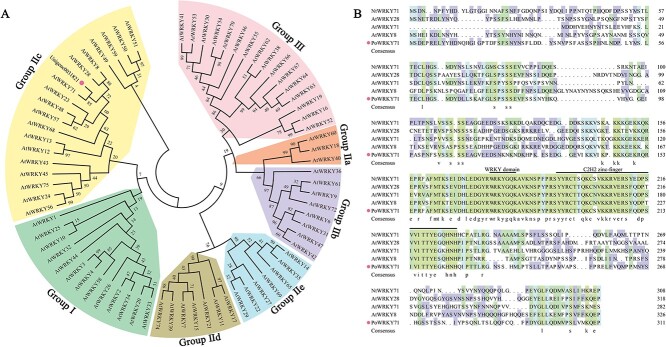
Phylogenetic analysis and amino acid sequence alignment of PoWRKY71. (**A**) Phylogenetic tree analysis of PoWRKY71 and AtWRKYs from *Arabidopsis thaliana.* PoWRKY71 is indicated with a dot. The full length amino acid sequences were downloaded from *A. thaliana* public TAIR database. (**B**) Comparative sequence analysis of PoWRKY71, AtWRKYs from *A. thaliana* and *Nicotiana tabacum* NtWRKY71. PoWRKY71 is indicated with a dot. The conserved regions are marked with solid lines.

**Figure 2:**
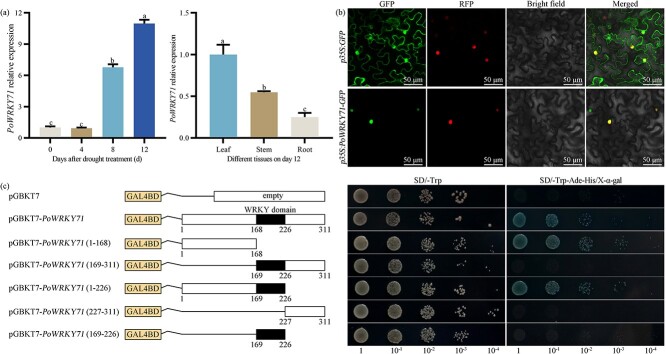
Expression characteristics of PoWRKY71. (**A**) Spatiotemporal expression profile of *PoWRKY71*. (**B**) Subcellular localization of PoWRKY71 in tobacco leaves. The nucleus localization mCherry protein was coinfiltrated with *p35S:GFP* and *p35S:PoWRKY71-GFP.* The GFP and RFP signals were excited and visualized with 488 nm and 561 nm lasers, respectively. (**C**) Transcriptional activation of PoWRKY71 in yeast system. The full-length of PoWRKY71 and five truncated fragments, 1–168 aa, 169–311 aa, 1–226 aa, 227–311 aa, and 169–226 aa were introduced into the pGBKT7 vector, and transgenic yeast cells were plated on SD/−Trp and SD/−Trp-Ade-His/X-α-gal medium for transcriptional activation activity detection.

To dig drought-responsive genes, we previously conducted transcriptome analysis on *P. ostii* leaves between the controls and 12 days after drought treatment [24] and mined a drought-triggered WRKY transcription factor with significantly up-regulated expression in leaves after 12 days of treatment. The coding sequence (CDS) of Unigene0031821 was 936 bp, which encoded 311 aa protein. The phylogenetic tree containing *A. thaliana* WRKY family members divided Unigene0031821 into Group IIc. It demonstrated that Unigene0031821 obtained the most similarity to AtWRKY71, which was initially named PoWRKY71 (Fig. 1A). The protein sequence analysis showed that PoWRKY71 contained a conserved WRKY domain and a zinc finger (Fig. 1B). The response of *PoWRKY71* to drought treatment was initially verified by the expression levels in *P. ostii* leaves on 0, 4, 8, and 12 days after drought treatment. The expression of *PoWRKY71* showed no change on day 4 but increased rapidly on day 8 and day 12, which reached 6.77 and 10.98 times that of day 0. The spatial expression results showed that *PoWRKY71* was specifically expressed in leaves on day 12 after drought treatment, which was 1.82 and 4.00 times higher than that in stems and roots (Fig. 2A). PoWRKY71 was localized in the nucleus of *Nicotiana benthamiana* epidermal cells (Fig. 2B), and protein truncated analysis showed that its activation domain was located in N-terminal and its transcriptional activity was independent of WRKY domain (Fig. 2C). These results indicated that *PoWRKY71* was a transcriptional activator induced by drought stress.

### Heterologous overexpression of *PoWRKY71* improves tolerance to drought stress

**Figure 3:**
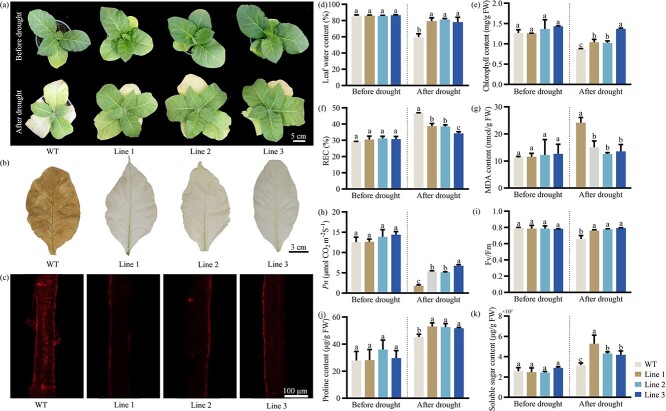
Effect of drought stress on the wild-type and *PoWRKY71*-overexpressing plants. (**A**) Phenotype observation between wild-type and *PoWRKY71*-overexpressing plants on days 0 and 15 of drought treatment. (**B**) H_2_O_2_ accumulation when *PoWRKY71* was overexpressed. (**C**) O_2_^·−^ accumulation when *PoWRKY71* was overexpressed. (**D**) Leaf water content when *PoWRKY71* was overexpressed. (**E**) Chlorophyll content when *PoWRKY71* was overexpressed. (**F**) REC when *PoWRKY71* was overexpressed. REC, relative electric conductivity. (**G**) MDA content when *PoWRKY71* was overexpressed. MDA, malondialdehyde. (**H**) *Pn* when *PoWRKY71* was overexpressed. *Pn*, net photosynthetic rate. (**I**) *F_v_/F_m_* when *PoWRKY71* was overexpressed. *F_v_/F_m_*, photochemical efficiency. (**J**) Proline content when *PoWRKY71* was overexpressed. (**K**) Soluble sugar content when *PoWRKY71* was overexpressed. WT, wild-type.

To verify the potential function of *PoWRKY71* in drought stress, *PoWRKY71* was heterologously overexpressed in *Nicotiana tabacum*, and PCR was conducted to identify positive transgenic lines (Fig. S1, see online supplementary material). Under normal growth conditions, the chlorophyll content and *Pn* in *PoWRKY71* transgenic lines showed no significant difference with wild-type (WT). When they were exposed to the drought treatment, WT revealed largely inhibited growth and the leaves gradually wilted, while the transgenic lines still maintained normal growth and showed stronger drought tolerance (Fig. 3A). The survival rate of *PoWRKY71* transgenic lines increased by an average of 20.32% (Fig. S2, see online supplementary material). Moreover, the accumulation of H_2_O_2_ and superoxide anion free radical (O_2_^·−^) in *PoWRKY71* transgenic lines was much lower than in WT under drought treatment (Fig. 3B and C). Consistently, the leaf water content and chlorophyll content decreased after drought, but the decline was much lower in *PoWRKY71* transgenic lines than those in WT (Fig. 3D and E). The *PoWRKY71* transgenic lines all showed less rise in relative electric conductivity (REC) and MDA content than in WT, whereas the photosynthesis-related *Pn* and photochemical efficiency (*F_v_/F_m_*) maintained higher levels (Fig. 3F–I). In addition, the activities of four protective enzymes, proline and soluble sugar content were measured, and they all abundantly increased and accumulated in *PoWRKY71* transgenic lines (Fig. 3J and K; Fig. S3, see online supplementary material). Obviously, overexpression of *PoWRKY71* notably improved plant tolerance to drought stress.

### Silencing of *PoWRKY71* in *P. ostii* increases sensitivity to drought stress

**Figure 4:**
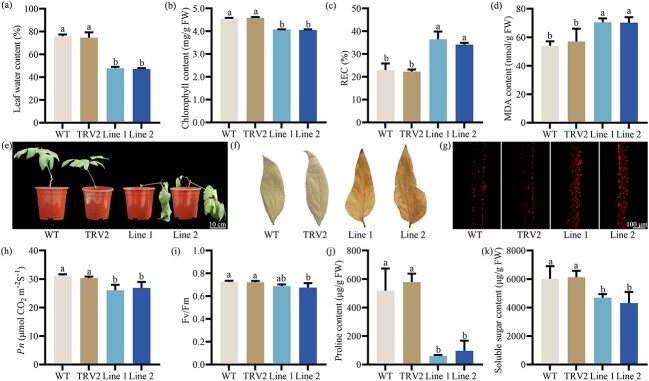
Effect of drought stress on the wild-type and *PoWRKY71*-silenced plants based on VIGS. (**A**) Leaf water content when *PoWRKY71* was silenced. (**B**) Chlorophyll content when *PoWRKY71* was silenced. (**C**) REC when *PoWRKY71* was silenced. REC, relative electric conductivity. (**D**) MDA content when *PoWRKY71* was silenced. MDA, malondialdehyde. (**E**) Phenotype of the wild-type and *PoWRKY71*-silenced plants after 15 days of drought treatment. (**F**) H_2_O_2_ accumulation when *PoWRKY71* was silenced. DAB, diaminobenzidine. (**G**) O_2_^·−^ accumulation when *PoWRKY71* was silenced. (**H**) *Pn* when *PoWRKY71* was silenced. *Pn*, net photosynthetic rate. (**I**) *F_v_/F_m_* when *PoWRKY71* was silenced. *F_v_/F_m_*, photochemical efficiency. (**J**) Proline content when *PoWRKY71* was silenced. (**K**) Soluble sugar content when *PoWRKY71* was silenced. WT, wild-type; TRV2, empty vector.

To further investigate the function of *PoWRKY71* in *P. ostii*, the transcription of *PoWRKY71* was disturbed by mRNA interference vectors based on virus-induced gene silencing (VIGS) technology. Plants were then subjected to drought. PCR amplification results confirmed that the TRV1 and TRV2 vectors were successfully transferred to *P. ostii*, and the expression level of *PoWRKY71* was largely inhibited by 66.88% (Fig. S4, see online supplementary material). After drought treatment, the leaf water content in *PoWRKY71*-silenced plants decreased by 37.38% compared to WT (Fig. 4A). Considering the significantly stable chlorophyll content in leaves of the *PoWRKY71* overexpressing lines after drought treatment, we evaluated the chlorophyll content in *PoWRKY71*-silenced plants. As expected, silencing *PoWRKY71* accelerated the degradation of chlorophyll (Fig. 4B).

Light-harvesting chlorophyll a/b-binding (CAB) protein is an important pigments protein that forms a complex with chlorophyll in plants, which promotes photosynthesis by regulating light capture and transfer, and CAB may also function in resisting environmental stress [33]. Logically, we evaluated the expression level of *PoCAB151* gene in *PoWRKY71*-silenced plants [24]. Under the restriction of *PoWRKY71*, *PoCAB151* gene expressions were significantly suppressed by an average decrease of 33.90% (Fig. S5, see online supplementary material). In contrast to *PoWRKY71* overexpressing lines, the *PoWRKY71*-silenced lines accumulated multiple H_2_O_2_ and O_2_^·−^, and the REC and MDA content increased remarkably, which was consistent with the phenotype observations (Fig. 4C–G). Meanwhile, *PoWRKY71*-silenced lines showed reduced *Pn*, *F_v_/F_m_*, protective enzyme activities, and proline and soluble sugar content (Fig. 4H–K; Fig. S6, see online supplementary material). Collectively, these results indicated that *PoWRKY71* positively responded to drought stress in *P. ostii*.

### PoWRKY71 directly activates the transcription of *PoCAB151*

To initially study the function of *PoCAB151* in drought-stress response, the CDS of *PoCAB151* was cloned, which was 792 bp in length and encoded 264 aa protein. The phylogenetic tree and sequence analysis identified that *PoCAB151* was highly conserved as CAB151 proteins from other species and had a typical chlorophyll A–B binding domain in the N-terminal (Fig. S7A and B, see online supplementary material). The subcellular localization of PoCAB151 was determined by PoCAB151-GFP fusion vector, and green fluorescent protein (GFP) signals were overlapped with chloroplast autofluorescence, indicating that PoCAB151 was a typical chloroplast protein (Fig. S7C, see online supplementary material).

**Figure 5:**
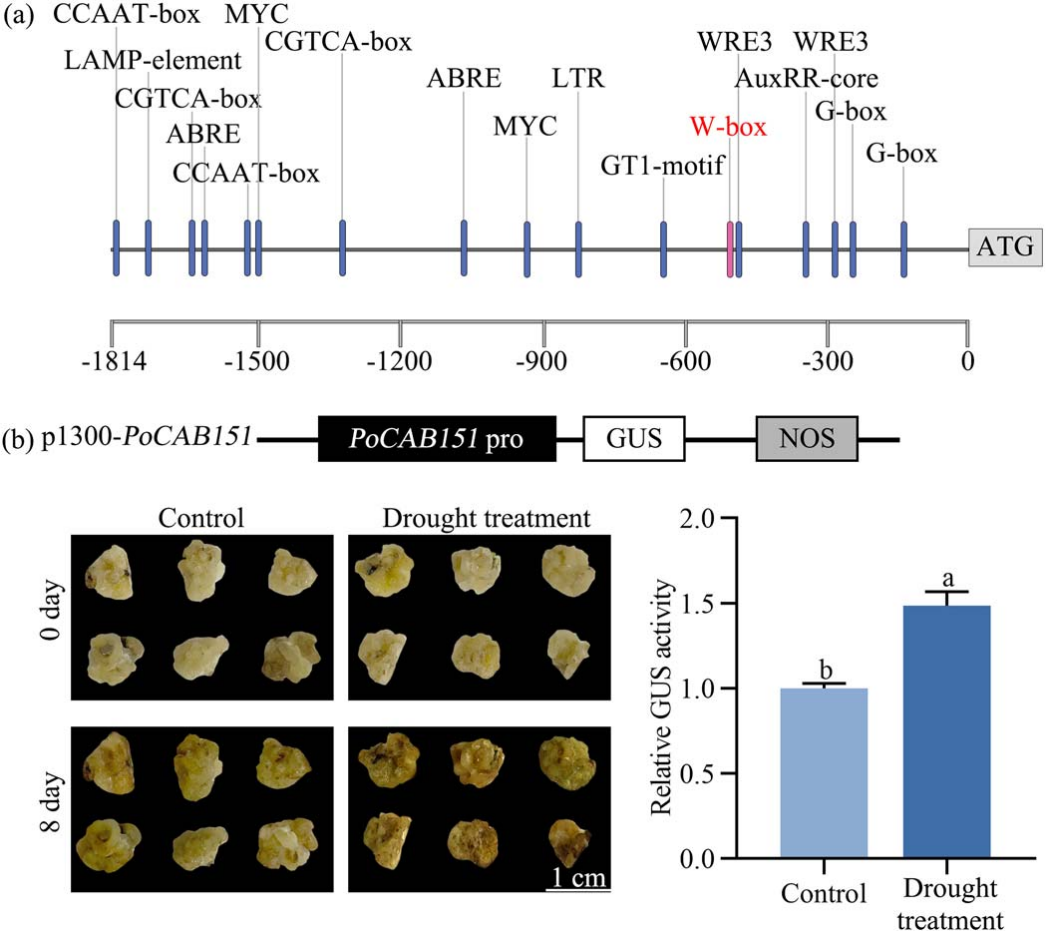
Isolation of *PoCAB151* promoter and its response to drought stress. (**A**) *cis*-elements in the *PoCAB151* promoter. A 1814 bp length of *PoCAB151* promoter was used for analysis by online PlantCARE database. The core elements in *PoCAB151* promoter were marked in its corresponding sites. (**B**) β-glucuronidase (GUS) activity analysis of the *PoCAB151* promoter in transgenic *Paeonia ostii* callus under drought treatment using WPM medium containing 10% PEG 6000. The *PoCAB151* promoter was introduced into the GUS reporting vector, and then transiently overexpressed in *P. ostii* callus. The overexpressed *P. ostii* callus was cultured on WPM and WPM containing 10% PEG 6000 for 8 days until GUS activity analysis was performed to judge whether the *PoCAB151* promoter respond to drought stress. WPM, woody plant medium.

To test *PoCAB151* response to drought, a 1814 bp length region upstream from the *PoCAB151* gene was obtained. PlantCARE analysis results revealed that *PoCAB151* promoter contained essential light-responsive elements including GT1-motif, G-box, and LAMP elements (Fig. 5A). A β-glucuronidase (GUS) reporter system was introduced to examine *PoCAB151* promoter activity in drought-treated *P. ostii* callus. After 8 days of treatment on drought simulated medium, transgenic *P. ostii* callus overexpressing *PoCAB151pro* exhibit higher GUS activity (a 48.63% increase) compared with the control group (without drought treatment) (Fig. 5B).

**Figure 6:**
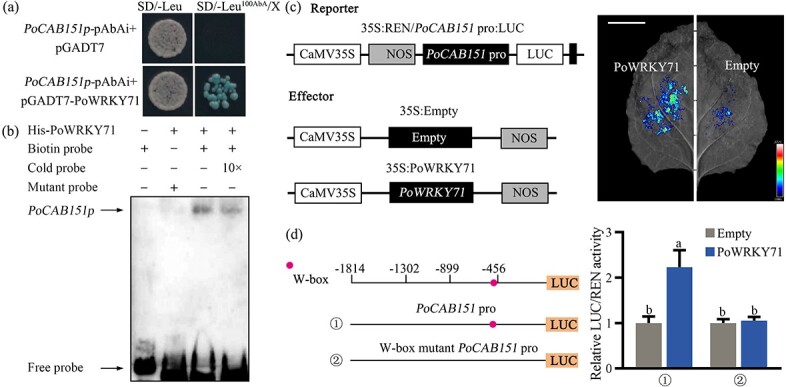
PoWRKY71 activated the promoter of *PoCAB151*. (**A**) The interaction relationship between PoWRKY71 and *PoCAB151* analysed by yeast one-hybrid (Y1H) assay. The Y1H Gold cells harbouring pGADT7-PoWRKY71 prey plasmids and *PoCAB151-*pAbAi bait plasmids were selected on SD/−Leu^100AbA^ medium. X, X-α-gal. (**B**) The interaction relationship between PoWRKY71 and *PoCAB151* analysed by electrophoretic mobility shift assay. The biotin probes are 34 bp *PoCAB151* promoter fragments containing a W-box element, and the cold probe without biotin labels were used as a binding competitor. (**C**) The interaction relationship between PoWRKY71 and *PoCAB151* analysed by luciferase reporter assay. The luciferase activity of the tobacco leaf was imaged after infiltration with 1 mM D-luciferin potassium salt. Bar = 5 cm. (**D**) Activation effects of PoWRKY71 on full-length and W-box mutant *PoCAB151* promoters. The W-box element is indicated with a dot.

Based on the down-regulated expression of *PoCAB151* in *PoWRKY71*-silenced plants and the positive response of *PoCAB151* promoter to drought stress, we hypothesized that there might have existed an interactive relationship between PoWRKY71 and *PoCAB151*. To further study the relationship between PoWRKY71 and *PoCAB151*, yeast one-hybrid (Y1H) assay was first performed, and PoWRKY71 was defined as a prey protein. As shown in Fig. 6A, all yeasts could grow on the SD/−Leu medium, while only PoWRKY71 could trigger the activity of the *PoCAB151* promoter, thus making the PoWRKY71-*PoCAB151* transgenic yeasts grow normally on the SD/−Leu medium containing Aureobasidin A (AbA), which suggested that PoWRKY71 could bind to the promoter of *PoCAB151*. Electrophoretic mobility shift assay (EMSA) was carried out to further identify the core binding sequence of PoWRKY71 to the *PoCAB151* promoter, and gel-shift assay indicated that His-PoWRKY71 fusion protein could form a complex with the biotin DNA probes containing the W-box element, but could not bind to W-box mutant probes (Fig. 6B). The regulatory effect of PoWRKY71 on the *PoCAB151* promoter was further tested by the luciferase reporter assay (LRA), and the fluorescence intensity was used to judge the degree of PoWRKY71 regulation on the *PoCAB151* promoter. After spraying luminescent substrate, substantial fluorescence signals were detected on the side impregnated with PoWRKY71, while only a small amount of fluorescence signals was detected on the empty vector side, and the firefly luciferase (LUC)/*Renilla* luciferase (REN) activity of *PoCAB151* promoter with PoWRKY71 presence was almost two times than that of the empty vector, while PoWRKY71 had no effect on the activity of the W-box element mutant *PoCAB151* promoter (Fig. 6C and D). Taken together, we found that PoWRKY71 could directly activate the transcription of *PoCAB151* by targeting the W-box.

### Heterologous overexpression of *PoCAB151* enhances plant tolerance to drought stress

**Figure 7:**
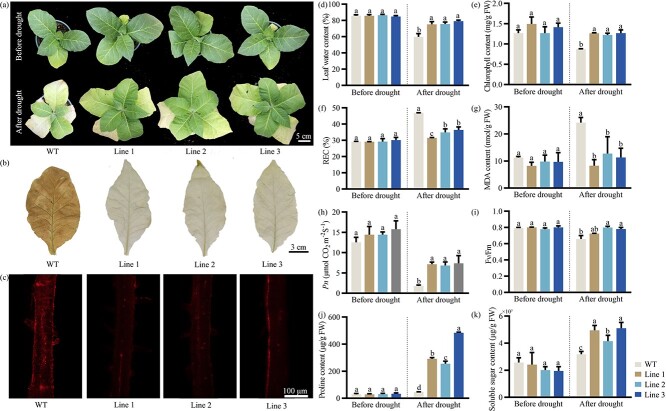
Effect of drought stress on the wild-type and *PoCAB151*-overexpressing plants. (**A**) Phenotype of the wild-type and *PoCAB151*-overexpressing plants on day 0 and 15 of drought treatment. (**B**) H_2_O_2_ accumulation when *PoCAB151* was overexpressed. (**C**) O_2_^·−^ accumulation when *PoCAB151* was overexpressed. (**D**) Leaf water content when *PoCAB151* was overexpressed. (**E**) Chlorophyll content when *PoCAB151* was overexpressed. (**F**) REC when *PoCAB151* was overexpressed. REC, relative electric conductivity. (**G**) MDA content when *PoCAB151* was overexpressed. MDA, malondialdehyde. (**H**) *Pn* when *PoCAB151* was overexpressed. *Pn*, net photosynthetic rate. (**I**) *F_v_/F_m_* when *PoCAB151* was overexpressed. *F_v_/F_m_*, photochemical efficiency. (**J**) Proline content when *PoCAB151* was overexpressed. (**K**) Soluble sugar content when *PoCAB151* was overexpressed. WT, wild-type.

To further clarify the relationship between *PoCAB151* and drought tolerance, the function of *PoCAB151* was more directly investigated by heterologous transformation experiments in tobaccos. The positive *PoCAB151* transgenic lines were first validated by PCR (Fig. S8, see online supplementary material). Under normal growth conditions, the chlorophyll content and *Pn* in *PoCAB151* transgenic lines were slightly higher than in WT, but there was no significant difference. When exposed to drought treatment, the drought damage phenotype of WT was much more severe than *PoCAB151* transgenic lines with more H_2_O_2_ and O_2_^·−^ accumulation (Fig. 7A–C). In deep, the physiological changes included higher leaf water content, chlorophyll content, *Pn*, *F_v_/F_m_*, proline and soluble sugar content, and lower REC and MDA content were identified in *PoCAB151* transgenic line (Fig. 7D–K). In addition, the survival rate of *PoCAB151* transgenic lines increased by an average of 18.06% (Fig. S9, see online supplementary material). Collectively, we speculated that overexpression of *PoCAB151* defended against drought stress by stabilising photosynthesis and promoted drought-resistant substance accumulation via regulating chlorophyll content in *P. ostii*.

### Loss of *PoCAB151* reduces *P. ostii* resistance to drought stress

**Figure 8:**
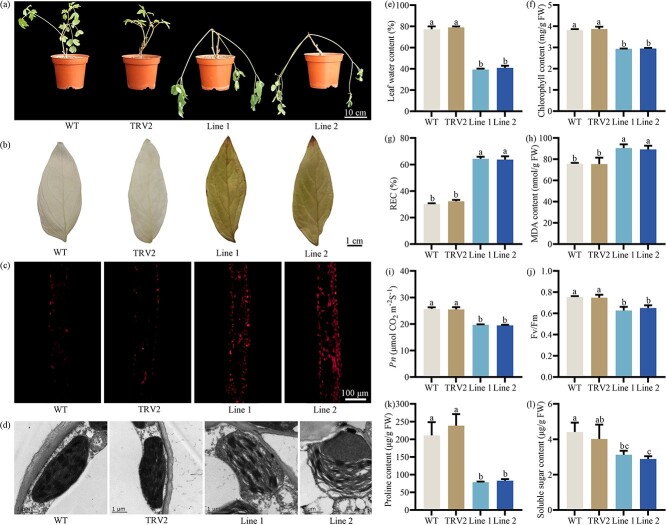
Effect of drought stress on the wild-type and *PoCAB151*-silenced plants based on VIGS. (**A**) Phenotype of the wild-type and *PoCAB151*-silenced plants after 15 days of drought treatment. (**B**) H_2_O_2_ accumulation when *PoCAB151* was silenced. (C) O_2_^·−^ accumulation when *PoCAB151* was silenced. (**D**) Leaf anatomical observation when *PoCAB151* was silenced. (**E**) Leaf water content when *PoCAB151* was silenced. (**F**) Chlorophyll content when *PoCAB151* was silenced. (**G**) REC when *PoCAB151* was silenced. REC, relative electric conductivity. (**H**) MDA content when *PoCAB151* was silenced. MDA, malondialdehyde. (**I**) *Pn* when *PoCAB151* was silenced. *Pn*, net photosynthetic rate. (**J**) *F_v_/F_m_* when *PoCAB151* was silenced. *F_v_/F_m_*, photochemical efficiency. (**K**) Proline content when *PoCAB151* was silenced. (**L**) Soluble sugar content when *PoCAB151* was silenced. WT, wild-type; TRV2, empty vector.

In addition, *PoCAB151* was successfully silenced in *P. ostii* via VIGS technology, and the expression of *PoCAB151* decreased by an average of 64.33% (Fig. S10, see online supplementary material). After exposure to drought treatment, *PoCAB151*-silenced plants showed greater drought intolerance and higher H_2_O_2_ and O_2_^·−^ accumulation, and the normal structure of chloroplast was destroyed with a high disintegration degree (Fig. 8A–D). Moreover, silencing of *PoCAB151* resulted in lower leaf water content, chlorophyll content, *Pn*, *F_v_/F_m_*, proline and soluble sugar content, and higher REC and MDA content (Fig. 8E–L). The above results demonstrated that *PoCAB151* actively stabilised photosynthesis via regulating chloroplast homeostasis and chlorophyll content, and promoted drought-resistant substance accumulation to positively respond to drought stress.

## Discussion

### Drought-stress-responsive *PoWRKY71* plays a positive role in drought resistance

WRKY transcription factors are plant-specific transcription factors, and genome-wide analysis annotated 119 *ZmWRKYs* in *Z. mays*, 109 *OsWRKYs* in rice, and 182 *GmWRKYs* in soybean [34–36]. WRKY transcription factors are partly framed by the conserved WRKY domain and zinc-finger motif [37]. Over the past few decades, WRKY transcription factors were verified to participate in a series of abiotic stress as positive or negative regulators including high temperature, cold, drought, salt, and oxidative stresses [18]. Drought stress usually induces WRKY transcription factor expression and then triggers the network hierarchy of plant defenses [38]. For instance, 32 *Elaeis guineensis EgWRKY* transcription factors exhibited higher expression levels during drought treatment [39]. A genetic study revealed that *Triticum aestivum TaWRKY1* and *TaWRKY33* enhance transgenic *Arabidopsis* plants’ drought resistance [40]. *P. ostii* is an important woody oil crop, and the unsaturated fatty acids in its seeds have great benefits to human health [22], while few of the literature focused on drought stress research that affects its planting expansion. Especially, the roles of WRKY transcription factors in the *P. ostii* drought resistance are largely unknown and have never been elucidated. Based on the evolutionary non-conservation and functional diversity of regulatory proteins across different species, we previously performed transcriptome analysis to dig drought-responsive WRKY transcription factors. Here, a differentially expressed *PoWRKY71* gene that was highly expressed in drought-treated *P. ostii* was screened through transcriptome analysis and quantitative real-time PCR (qRT-PCR) validation, and sequence analysis defined PoWRKY71 as a Group IIc member of the WRKY family. *A. thaliana* AtWRKY71, which was most relative to PoWRKY71 was verified to facilitate shoot branching and leaf senescence [41, 42], whereas *P. ostii PoWRKY71* was initially identified as a novel drought-responsive gene due to its corresponding transcript levels. *PoWRKY71* was specifically expressed in the leaves of drought-treated *P. ostii*, and its transcript level gradually increased with the degree of drought. Moreover, PoWRKY71 demonstrated transcriptional activation activity dependent on its activation domain in N-terminal, which showed the same results as *Fragaria vesca* FvWRKY71 [43]. In this study, overexpressing *PoWRKY71* in tobacco greatly improved plant tolerance to drought stress, whereas silencing *PoWRKY71* in *P. ostii* remarkably decreased plant tolerance. All these results verified that *PoWRKY71* actively promoted drought resistance in *P. ostii*.

As the most important physiological process, photosynthesis greatly limits plant growth and yield, and stress-related positive regulatory proteins always sense the upstream environmental signals and then participate in plant stress resistance processes, including regulating photosynthesis and chlorophyll content [44]. In transgenic *T. aestivum*, plants accumulated more chlorophyll to resist drought stress after overexpressing *Gossypium herbaceum GhDREB* gene [45]. Under drought treatment, all plants showed decreased photosynthesis and chlorophyll content, but the photosynthesis and chlorophyll content in *PoWRKY71* transgenic tobaccos were much higher than that in WT, together with higher drought tolerance. Conversely, *PoWRKY71*-silenced plants exhibited lower drought tolerance when compared with the WT, and drought treatment dramatically accelerated the degradation of chlorophyll. In the leaf senescence process of *Arabidopsis*, chlorophyll content decreased in *AtWRKY71* overexpressing plants and increased in *AtWRKY71* knockout plants, which was contrary to our results [42]. This might be attributed to the diversity of the evolutionary process of transcription factors in different species. Further evaluation captured that photosynthesis-related *PoCAB151* gene was significantly downregulated in *PoWRKY71*-silenced plants, implying *PoCAB151* might undergo the regulation of PoWRKY71 protein.

### *PoCAB151* regulated by PoWRKY71 participates in drought resistance

CAB proteins are highly conserved in evolution, and participate in the light capture and transfer in photosynthesis as well as play positive roles in adaption to external stresses [46]. Likewise, *PoCAB151* was highly conserved in structure with the typical chlorophyll A–B binding domain in the N-terminal as other species [47, 48]. Next, *PoCAB151* promoter activity was proven to increase in drought-treated *P. ostii* callus on Day 8, which was consistent with *PoWRKY71* expression changes. Then, Y1H experiment, EMSA, and LRA all verified that PoWRKY71 protein could specially recognize and bind to the W-box element of *PoCAB151* promoter and promote its transcription. The shreds of evidence confirmed the drought-induced PoWRKY71-*PoCAB151* pathway in *P. ostii*. *M. domestica* MdWRKY17 stabilized the normal growth of plants under drought stress by binding on the W-box of the chlorophyll biosynthesis-related *MdSUFB* gene promoter [44]. *G. herbaceum* GhWRKY1-like was reported to positively respond to drought stress by manipulating ABA biosynthesis-related *AtNCED2*/*5*/*6*/*9* genes in transgenic *Arabidopsis* [49]. *T. aestivum TaGAPC5* was directly regulated by TaWRKY28, TaWRKY33, TaWRKY40, and TaWRKY47 proteins, and increased drought tolerance by accelerating ROS scavenging and stomatal movement [50]. Here, multiple pieces of evidence uncover the PoWRKY71-*PoCAB151* regulatory pathway in *P. ostii* under drought stress, whereas the specific function of *PoCAB151* needs to be further investigated.

### *PoCAB151* positively responds to drought stress by regulating photosynthesis and chlorophyll content

When plants encounter drought stress, a range of changes including stomatal regulation, root development, and hormonal responses are initiated to cope with this damage. Here, it is urgent to explore the regulatory mechanism of how the PoWRKY-*PoCAB151* pathway increases plant tolerance under drought stress. When the *CAB* gene was concerned, previous studies have displayed its response to abiotic stress in several species as a highly abundant membrane protein, and its expression is always affected by abiotic stresses [51, 52]. In *A. thaliana*, *CAB* family members respond to drought stress via modulating ROS homeostasis [33]. Overexpressing *M. domestica MdLHCB4.3* enhanced drought tolerance of transgenic apple callus [47]. Similar to our present study, both overexpression and VIGS experiments of *PoCAB151* confirmed the involvement of *PoCAB151* in stabilising photosynthesis via regulating chlorophyll content. Notably, chloroplast disintegration was observed in *PoCAB151*-silenced plants, further demonstrating that *PoCAB151* positively regulated to drought tolerance by maintaining the homeostasis of the photosynthesis site. Overexpression of *Actinidia chinensis AcLHCB3.1* and *AcLHCB3.2* resulted in a remarkable increase of chlorophyll a content in tobacco leaves [53], and our results further demonstrate the association between photosynthesis, chloroplast homeostasis and chlorophyll with drought tolerance. In plants, proline and soluble sugar are important osmotic protectants defending drought stress [54, 55]. As in our study, both proline and soluble sugar substantially accumulated in *PoCAB151* overexpressing plants, while their contents in *PoCAB151*-silenced plants were much lower than in WT and empty vector. This suggested that *PoCAB151* could promote the accumulation of drought-resistant substances to resist drought, and a similar result was verified in *M. domestica* [47]. Van Aken *et al.* [56] has shown that *A. thaliana LHCB2.4* gene expression was inhibited by AtWRKY57 during initial stress response to plants, while our results suggested that another WRKY transcription factor family member PoWRKY71 functioned positively in *P. ostii* drought resistance by activating *PoCAB151* expressions, which indicated that the regulatory network of drought resistance may be different between species.

**Figure 9:**
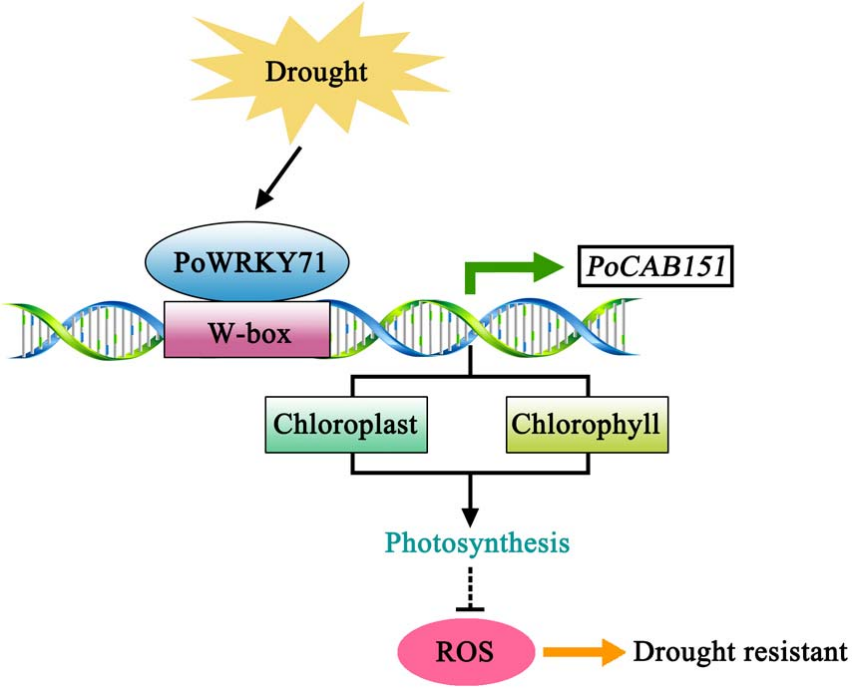
A proposed model for PoWRKY71-*PoCAB151* regulatory pathway during drought resistance in *P. PoWRKY71* was induced by drought stress, which subsequently activated *PoCAB151* transcription by binding on the W-box element. *PoCAB151* stabled photosynthesis via regulating chloroplast homeostasis and chlorophyll content to eliminate over-accumulated ROS, thus enhancing plant resistance to drought stress.

All in all, we demonstrated that a WRKY transcription factor PoWRKY71 was isolated and characterized from *P. ostii*. *PoWRKY71* specifically expressed in drought-treated *P. ostii* and functional experiments verified the positive role of *PoWRKY71* under drought stress. PoWRKY71 targeted the W-box element on *PoCAB151* promoter, and *PoCAB151* stabilised photosynthesis via regulating chloroplast homeostasis and chlorophyll content under drought stress. Furthermore, numerous clues showed that *PoCAB151* could eliminate the over-accumulation of ROS and endowed plants with high drought resistance (Fig. 9). This study not only broadens our understanding of *P. ostii* drought resistance mechanism but also provides a feasible strategy for improving plant drought resistance via stabilising photosynthesis.

## Materials and methods

### Plant materials and growth conditions

One-year-old plants of *P. ostii* were grown in potting soil (loam/peat/perlite, 1:1:1) in a greenhouse. We carried out three days of continuous watering before the drought treatment and then exposed plants to natural drought without water. Leaves, roots, and stems were collected at the designated time referring to the previous study [24]. *N. tabacum* and *N. benthamiana* were grown in a plant growth chamber (25°C 16 h light / 22°C 8 h dark). To generate *N. tabacum* overexpression lines, the CDS of *PoWRKY71* and *PoCAB151* were fused into pB1301 (driven by maize polyubiquitin gene promoter) and pCAMBIA1301 vector (driven by cauliflower mosaic virus [CaMV] 35S promoter) with gene-specific primers (Table S1, Fig. S11, see online supplementary material). Leaf disc method was applied here to obtain transgenic tobacco according to that described in Sunilkumar *et al.* [57]. The drought treatment of plants in overexpression and VIGS assays were conducted as above and samples were collected at designated time points. *N. benthamiana* was used for subcellular location observation and LRA. The *P. ostii* callus was induced by leaves at leaf expansion stage and then cultured on woody plant medium (WPM) at 20°C in the dark for two months. To generate *PoCAB151* overexpressing *P. ostii* callus, the promoter of *PoCAB151* was fused into pCAMBIA1300 vector containing a GUS reporting system (Table S1, see online supplementary material), and the *Agrobacterium* containing *PoCAB151* promoter was used for infection. For drought treatment, the transgenic *P. ostii* callus were treated on the WPM containing 10% polyethylene glycol (PEG) 6000 and incubated at 20°C for 8 days. The untreated *P. ostii* callus was defined as the controls.

### DNA and RNA extraction, cDNA synthesis, and qRT-PCR analysis

Genomic DNA and total RNA extraction of *P. ostii* roots, stems, leaves, *N. tabacum* leaves and RNA reverse transcription were performed according to the established system [58]. qRT-PCR analysis was performed by collecting SYBR fluorescence with gene-specific primers. Gene relative expression was obtained using the 2^-△△Ct^ (comparative threshold cycle) formula. *P. ostii Ubiquitin* (JN699053) and *N. tabacum Actin* (AB158612) were used for normalization as the internal controls. All analyses consisted of three biological replicates. The primers employed here were shown in Table S1 (see online supplementary material).

### Gene, promoter cloning, and bioinformatic analysis

The CDS of *PoWRKY71* (Unigene0031821) and *PoCAB151* (Unigene0026374) were amplified from *P. ostii* leaves by gene-specific primers designed based on the previous *P. ostii* transcriptome database (SRA: SRP161474). PoWRKY71 and PoCAB151 homologous proteins from *A. thaliana* and other plants were downloaded from the NCBI database. MEGA7.2, DNAMAN 6.0, and MEME Suite 5.4.1 (https://meme-suite.org/) were used to dissect protein affinities. The promoter sequence of *PoCAB151* was cloned by the chromosome walking method. Potential *cis*-elements in the *PoCAB151* promoter were predicted by the online PlantCARE database (http://bioinformatics.psb. ugent.be/webtools/plantcare/html/) [59]. The primers employed here are shown in Table S1 (see online supplementary material).

### Subcellular localization of *PoWRKY71* and *PoCAB151*

For the subcellular localization of *PoWRKY71* and *PoCAB151*, CDSs were fused into the pCAMBIA2300 vector (driven by CaMV 35S promoter) (Table S1, Fig. S12, see online supplementary material). Overnight *Agrobacterium* cultures containing pCAMBIA2300-*PoWRKY71*, pCAMBIA1301-*PoCAB151*, and empty vectors were used to infiltrate *N. benthamiana* leaves. The GFP and RFP exciting at 488 nm 561 nm were observed by confocal laser microscopy (Nikon C2-ER, Tokyo, Japan).

### Transcriptional activation activity assay of *PoWRKY71*

*PoWRKY71* full length CDS and five truncated regions including 1–168 aa, 169–311 aa, 1–226 aa, 227–311 aa, and 169–226 aa were fused into the GAL4 DNA-binding domain of pGBKT7 vector [driven by alcohol dehydrogenase 1 (ADH1) gene promoter] with gene-specific primers (Table S1, see online supplementary material). Transgenic yeast strain AH109 expressing *PoWRKY71* protein was incubated onto the SD/−Trp and SD/−Trp-Ade-His/X-α-gal plates at 30°C for 48 h, and the growth and α-galactosidase staining conditions of the diluted yeast determined whether transcriptional activation activities existed.

### Overexpression of *PoWRKY71* and *PoCAB151* in tobacco

After transplanting rooted WT and transgenic tobaccos (*PoWRKY71* and *PoCAB151* overexpression) for two months, natural drought treatment was started after three days of continuous watering. Before drought treatment, plants with a similar phenotype were subjected to determination of leaf water content, chlorophyll content, photosynthetic characteristics, and chlorophyll fluorescence parameters. Then, the relevant physiological indices including H_2_O_2_, O2^·−^, REC, MDA content, proline and soluble sugar content, and protective enzyme activity [SOD, POD, CAT, and ascorbate peroxidase (APX)] were measured. The same indicators were measured again after 15 days of drought treatment. The survival rate assay was performed as previously described, and we counted survived plants in each pot after 20 days of drought treatment to calculate the survival rate [60]. The primers employed here were shown in Table S1 (see online supplementary material).

The leaf water content was calculated using the following formula: (fresh weight − drought weight) / fresh weight. The photosynthesis rate and chlorophyll fluorescence parameters were measured as described by Zhao *et al.* [24]. The accumulation of H_2_O_2_ was observed by diaminobenzidine (DAB) staining. The accumulation of O2^·−^ was detected by 10 μM dihydroethidium, and the fluorescence signals of samples (excitation at 540 nm, emission at 590 nm) were captured with a fluorescence microscope. The leaf REC was determined as previously reported [61]. The chlorophyll content, MDA content, proline content, soluble sugar content, and protective enzyme activity measurement were performed by reagent kits (Suzhou Corning Biotechnology Co., Ltd, Suzhou, China).

### VIGS of *PoWRKY71* and *PoCAB151* in *P. ostii*

The TRV-based VIGS system was applied to silence *PoWRKY71* and *PoCAB151* in *P. ostii* [62]. Briefly, the non-conserved fragments of *PoWRKY71* (from 4 to 288 bp) and *PoCAB151* (from 1 to 163 bp) were inserted into TRV2 vector (Fig. S13, see online supplementary material). Overnight *Agrobacterium* cultures containing TRV2-*PoWRKY71*, TRV2-*PoCAB151*, TRV2, and TRV1 were resuspended in rejuvenation buffer for 1 h.

*P. ostii* plants with 2–3 buds were used as materials and the roots were cleaned with sterile water. Before infection, TRV2-*PoWRKY71*, TRV2-*PoCAB151*, and TRV2 were mixed with TRV1 in a 1:1 ratio (v/v), and the roots were infiltrated into the mixed bacterial solution and filtered under vacuum for 30 min. Followed by washing twice with sterilized water, the plants were then replanted in potting soil (loam/peat/perlite, 1:1:1). Each group included 15 plants. After 30 days of cultivation, the plants at leaf-expanding stage were subjected to natural drought stress. Leaves were collected for PCR and qRT-PCR validation and leaf anatomical observation after about 12 days of drought treatment [29]. Leaf water content, chlorophyll content, photosynthesis rate, chlorophyll fluorescence parameters, H_2_O_2_, O2^·−^, REC, MDA content, proline, soluble sugar content, and protective enzyme activity were also measured. The primers employed here are shown in Table S1 (see online supplementary material).

### GUS activity analysis

The transgenic *P. ostii* callus was used for GUS activity analysis via the ELISA method. Briefly, 0.1 g fresh *P. ostii* callus samples were extracted and subjected to an enzyme-linked reaction as previously described to calculate GUS activity [63]. All analyses consisted of three biological replicates.

### Y1H assay

*PoWRKY71* CDS was fused into the pGBKT7 vector (driven by alcohol ADH1 gene promoter) as a prey vector, and *PoCAB151* promoter was inserted into the pAbAi vector as a bait vector with gene-specific primers (Table S1, see online supplementary material). The protein-gene interaction assay was performed as Luan *et al.* [58] previously described.

### EMSA

*PoWRKY71* CDS was cloned into the pET-SUMO expression vector with a His tag and for prokaryotic expression of fusion His-tagged PoWRKY71 protein. The probes containing a W-box element and a mutant W-box element in the *PoCAB151* promoter were labeled with biotin, and the unlabeled probes were used as a binding competitor. EMSA between purified PoWRKY71 protein and the probes was carried out as reported in Luan *et al.* [58] The primers and probes employed here were shown in Table S1 (see online supplementary material).

### Luciferase reporter assay (LRA)

*PoWRKY71* CDS was fused into the pGreenII-62-SK vector (driven by CaMV 35S promoter) as effector plasmids. The full length and W-box mutant *PoCAB151* promoter was fused into the pGreenII-0800-LUC vector as reporter plasmids. The primers employed here were shown in Table S1 (see online supplementary material). Overnight *Agrobacterium* cultures were mixed (effector/ reporter, 10:1, v/v) to infect 4 to 5-week-old *N. benthamiana* leaves. After 2 days of weak light cultivation, the luciferase activity was visualized after spraying 1 mM D-luciferin potassium salt (Beyotime, Shanghai, China), as well as measured by a Dual-Luciferase Reporter Assay Kit (Vazyme, Nanjing, China).

### Statistical analysis

The SAS/STAT statistical analysis package (version 6.12, USA) was used for statistical analysis. All data were average values of three replicates with standard deviations, and means were considered statistically significant at *P* < 0.05.

## Supplementary Material

Supplementary_figures_uhad194

Supplementary_table_1_uhad194

qPCR_analysis_results_uhad194

## Data Availability

All relevant data in this study can be found in the article and its supporting files.
