# Supplementary material for: PoWRKY71 is involved in Paeonia ostii resistance to drought stress by directly regulating light-harvesting chlorophyll a/b-binding 151 gene
Source: Hortic Res. 2023 Sep 25;10(11):uhad194. doi: 10.1093/hr/uhad194 (PMC10673652; doi:10.1093/hr/uhad194)
Supplement: Supplementary_figures_uhad194 [file supplementary_figures_uhad194.zip › Supplementary_figures_uhad194.doc]

**Figure S1.** PCR Identification of *PoWRKY71* transgenic tobacco lines. Hyg, hygromycin; WT, wild-type.

**Figure S2.** The survival rate of the wild-type (WT) and *PoWRKY71* transgenic lines on days 0 and 20 of drought treatment. The values represent the means ± SDs, and different letters indicate significant differences (*P* < 0.05).

**Figure S3.** Four protective enzyme activities in the wild-type (WT) and *PoWRKY71*-overexpressing plants on days 0 and 15 of drought treatment. SOD, superoxide dismutase; POD, peroxidase; CAT, catalase; APX, ascorbate peroxidase. The values represent the means ± SDs, and different letters indicate significant differences (*P* < 0.05).

**Figure S4.** PCR and qRT-PCR identification of *PoWRKY71*‐silenced plants based on VIGS. WT, wild-type; TRV2, empty vector. The values represent the means ± SDs, and different letters indicate significant differences (*P* < 0.05).

**Figure S5.** Expression level of *PoCAB151* in *PoWRKY71*‐silenced plants. The values represent the means ± SDs, and different letters indicate significant differences (*P* < 0.05).

**Figure S6.** Four protective enzyme activities in the wild-type (WT) and *PoWRKY71*-silenced plants after 15 days of drought treatment. SOD, superoxide dismutase; POD, peroxidase; CAT, catalase; APX, ascorbate peroxidase. The values represent the means ± SDs, and different letters indicate significant differences (*P* < 0.05).

**Figure S7.** Characterization of PoCAB151.

(a) Phylogenetic tree analysis of PoCAB151 and CAB proteins from other species*.* PoCAB151 was indicated with a dot. The three highly conserved motifs were characterized with rectangle. The protein sequences were downloaded from NCBI and their GenBank IDs were as follows: NtCAB36 (XP_016468414.1, *Nicotiana tabacum*), NnCAB151 (XP_010270182.1 *Nelumbo nucifera*), McCAB (OVA20177.1 *Macleaya cordata*), FvCAB151 (XP_004293460.1 *Fragaria vesca* subsp. *vesca*), PdCAB151 (XP_034207560.1 *Prunus dulcis*), AtLHCB2 (AT2G05070.1 *Arabidopsis thaliana*). (b) Comparative sequence analysis of PoWRKY71 and AtWRKYs from *Arabidopsis thaliana.* PoCAB151 was indicated with a dot. The conserved chlorophyll A-B binding domain was marked with solid line. (c) Subcellular localization of PoCAB151 in tobacco leaves.

**Figure S8.** PCR Identification of *PoCAB151* transgenic tobacco lines. Hyg, hygromycin; WT, wild-type.

**Figure S9.** The survival rate of the wild-type (WT) and *PoCAB151* transgenic lines on days 0 and 20 of drought treatment. The values represent the means ± SDs, and different letters indicate significant differences (*P* < 0.05).

**Figure S10.** PCR and qRT-PCR identification of *PoCAB151*‐silenced plants based on VIGS. WT, wild-type; TRV2, empty vector. The values represent the means ± SDs, and different letters indicate significant differences (*P* < 0.05).

**Figure S11.** Schematic maps of vectors for tobacco transformation.

**Figure S12.** Schematic maps of vectors for subcellular localization.

**Figure S13.** Schematic maps of vectors for *P. ostii* plants transformation.
